# Multilingual character recognition dataset for Moroccan official documents

**DOI:** 10.1016/j.dib.2023.109953

**Published:** 2023-12-13

**Authors:** Ali Benaissa, Abdelkhalak Bahri, Ahmad El Allaoui

**Affiliations:** a Data Science and Competitive Intelligence Team (DSCI), ENSAH, Abdelmalek Essaadi University (UAE), Tetouan, Morocco; b Decisional Computing and Systems Modelling Team, Engineering Sciences and Techniques Laboratory, Faculty of Sciences and Techniques Errachidia, Moulay Ismail University of Meknes, Morocco; c Finance and Governance of Organizations team, Governance and Performance of Organizations laboratory, The National School of Management, Abdelmalek Essaadi University, Tangier, Morocco

**Keywords:** OCR dataset, Character recognition, Printed characters, Documents digitization, Moroccan documents, Moroccan characters images

## Abstract

This article focuses on the construction of a dataset for multilingual character recognition in Moroccan official documents. The dataset covers languages such as Arabic, French, and Tamazight and are built programmatically to ensure data diversity. It consists of sub-datasets such as Uppercase alphabet (26 classes), Lowercase alphabet (26 classes), Digits (9 classes), Arabic (28 classes), Tifinagh letters (33 classes), Symbols (14 classes), and French special characters (16 classes). The dataset construction process involves collecting representative fonts and generating multiple character images using a Python script, presenting a comprehensive variety essential for robust recognition models. Moreover, this dataset contributes to the digitization of these diverse official documents and archival papers, essential for preserving cultural heritage and enabling advanced text recognition technologies. The need for this work arises from the advancements in character recognition techniques and the significance of large-scale annotated datasets. The proposed dataset contributes to the development of robust character recognition models for practical applications.

Specifications Table

**Table:** 

Subject	Machine Learning (ML), Deep Learning (DL) and Computer Vision (CV)
Specific subject area	Digitize of the Moroccan official documents using artificial intelligence
Type of data	Images
How the data were acquired	The Moroccan official document dataset was generated through the utilization of a Python script combined with image processing techniques. The dataset creation process involved the programmatic application of a designated font to generate images that represented the characters.
Data format	Raw JPG images
Description of data collection	The data collection process involved obtaining the characters of Moroccan official documents using a Python script and image processing techniques. The script programmatically applied a designated font to generate representative characters. There were no specific conditions or factors under study, as the focus was on capturing a comprehensive range of characters used in Moroccan official documents. The data normalization process involved standardizing the image size and format to ensure consistency.
Data source location	· Institution: National School of Applied Science, L-SA, T-DSCI · City: Al Hoceima · Country: Morocco
Data accessibility	Repository name: Multilingual Character Recognition Dataset for Moroccan Official Documents Data identification number: doi:10.17632/xp3hrmywfm.1 Direct URL to data: https://data.mendeley.com/datasets/xp3hrmywfm Direct URL to the code: https://github.com/ali-Benaissa/character-images-generation/tree/main

## 1 Value of the Data

• This dataset is customized for Moroccan official documents, optimizing the digitization process. It significantly contributes to the efficient handling and processing of a wide array of languages commonly found within these documents.
• This dataset acts as a fundamental resource for researchers engaged in digitizing various languages, especially Moroccan official documents, and provides a starting point for those looking to digitize other diverse languages not covered within our dataset using our developed scripts.
• Diverse datasets contain Arabic, French, and Tamazight characters, aiding multilingual character recognition model development.
• It is valuable for training/testing systems detecting/interpreting characters in Moroccan official documents, due to the variety between the different characters presented in these datasets.
• Enables automated systems (e.g., OCR) to recognize characters from multiple languages in official documents.
• Dataset focus: Arabic, French, Tamazight, enhances character recognition and language processing.
• Foundation for expanding research and creating larger datasets covering more languages, fonts, and variations.

## 2 Objective

The aim of generating this dataset is to provide a valuable resource for researchers working on multilingual character recognition, specifically focusing on Arabic, French, and Tamazight languages, and those working on digitizing documents. The dataset aims to address the need for diverse and comprehensive character collections from these languages, which are commonly found in Moroccan official documents.

## 3 Data Description

To build the dataset, we embarked on a comprehensive process. Since standard fonts used in Moroccan official documents were not readily accessible on the internet, particularly for languages like Tifinagh and Arabic, we initiated the construction of raw datasets. The initial phase involved gathering the most commonly used fonts for each language. Subsequently, we built six distinct sub-datasets as follows:

• Alphabet: This dataset comprises lowercase and uppercase letters, covering the entire alphabet (a to z, A to Z).
• Digits: This dataset encompasses numbers from 0 to 9.
• Arabic: This dataset contains all the letters of the Arabic language.
• Tifinagh: The Tifinagh dataset includes all Tifinagh letters; those letters are using in Amazigh language.
• French Special Characters: This dataset focuses on special characters utilized in the French language, such as “à, é, ç, è...”.
• Symbols: The symbols dataset encompasses various symbols like “?”, “!”, “(”, “)” to facilitate data augmentation.

To ensure efficient organization and management, we structured the datasets in a hierarchical directory format. Table 1 provides an overview of the dataset structure, including samples, the number of classes, fonts number, images number of each character, total images, dimensions, and the directory structure for each sub-dataset. The main “DATA” directory houses subdirectories for each sub-dataset, allowing for easy retrieval and navigation of character images. Notably, character images within the dataset maintain a standardized resolution of 32×32 pixels, ensuring uniform dimensions, except the Arabic and Tifinagh characters are standardized at 64×64 pixels due to the intrinsic nature of these languages' letters, necessitating a higher resolution for accurate representation. This dataset was developed to encompass the essential elements present in Moroccan official documents, ensuring comprehensive coverage. Within these sub-datasets, individual fonts were employed to generate a multifaceted representation of characters. The “Fonts number” in Table 1 indicates the total count of distinct font types utilized in constructing the dataset. Each font variant contributes to the dataset's richness, catering to the nuanced typographic diversity encountered in these documents. This careful selection of fonts underscores the dataset's utility in accommodating variations, thereby promoting more robust character recognition models.

**Table 1:** Dataset.

## 4 Experimental Design, Materials and Methods

Font families represent a rich tapestry of typographic styles, essential in rendering text across various languages and scripts. These families encompass diverse typefaces meticulously designed to accurately present and recognize characters within specific writing systems. Table 2 presents a selection of few a font family examples used for each dataset.

**Table 2:** Examples of free-to-use fonts by font families for diverse sub-datasets.

Sub-datasets	Language/Script	Font family	Specific fonts
Uppercase Alphabet	Latin	Arial, Helvetica, Roboto	Arial Bold, Helvetica Bold, Roboto Regular
Lowercase Alphabet	Latin	Times New Roman, Open Sans	Times New Roman Italic, Open Sans Bold
Digits	Numerals	Calibri, Lato, Arial	Calibri Bold, Lato Light, Arial Black
Arabic	Arabic	Amiri, Droid Arabic Noto	Amiri Bold, Droid Arabic Noto Regular
Tifinagh	Tifinagh	Tifinagh, Lateef, Scheherazade	Tifinagh Abudrar Light, Lateef Regular, Scheherazade Bold
Symbols	Various	Segoe UI Symbol, Font Awesome	Segoe UI Symbol Regular, Font Awesome Solid
French special characters	Latin	Open Sans, Montserrat	Open Sans Italic, Montserrat Regular

**Fig. 1:**
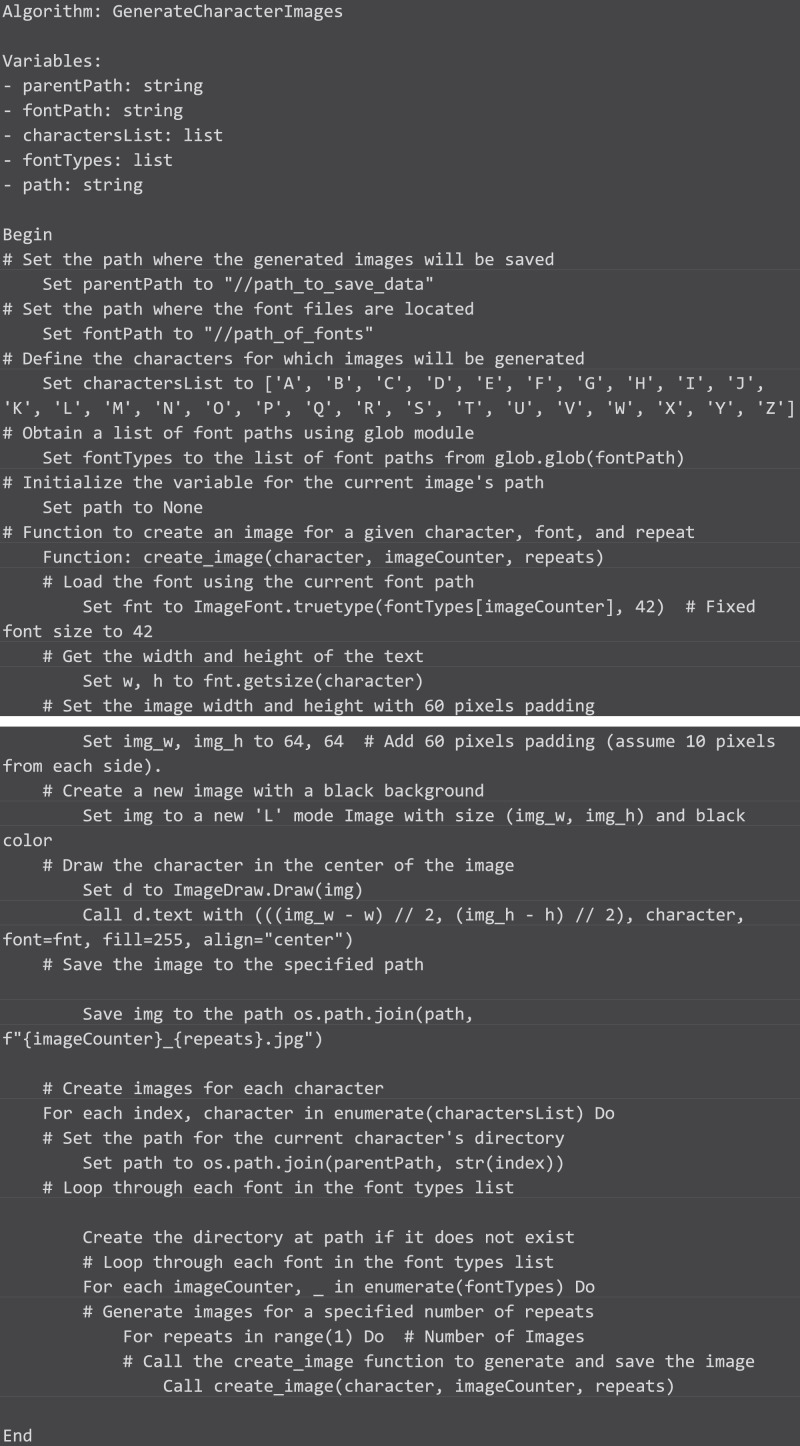
Algorithm.

To construct the dataset, we developed a custom Python [1] script. This script generated images for each character using the collected fonts. The algorithm for image generation is depicted in Fig. 1, each line in the accompanying the algorithm has been explained with comments, denoted by the '#' symbol above of each line.. The generated images exhibit a black background with white characters, minimizing distortions caused by lighting or shadows typically present in scanned images.

## 5 Conclusion

The creation of diverse and comprehensive datasets for character recognition in Moroccan official documents through a programmatically structured approach has resulted in a substantial resource for multilingual text analysis. These datasets encompass a wide array of languages, including Arabic, French, and Tifinagh, establishing a robust foundation for the development and evaluation of character recognition models. The meticulously organized datasets, with their defined parameters and diverse language coverage, not only contribute significantly to the domain of character recognition but also lay the groundwork for future research and innovations in Moroccan official document digitizing.

## Ethics Statements

The used fonts are collected from the internet, which are open source and available for download.

## CRediT authorship contribution statement

**Ali Benaissa:** Data curation, Methodology, Software, Writing – original draft, Visualization. **Abdelkhalak Bahri:** Conceptualization, Investigation, Writing – review & editing, Supervision, Project administration. **Ahmad El Allaoui:** Writing – review & editing.

## Data Availability

Multilingual Character Recognition Dataset for Moroccan Official Documents (Original data) (Mendeley Data)
